# Aggression and Food Resource Competition between Sympatric Hermit Crab Species

**DOI:** 10.1371/journal.pone.0091823

**Published:** 2014-03-14

**Authors:** Mark V. Tran, Matthew O’Grady, Jeremiah Colborn, Kimberly Van Ness, Richard W. Hill

**Affiliations:** 1 Department of Zoology, Michigan State University, East Lansing, Michigan, United States of America; 2 Ecology, Evolutionary Biology, and Behavior Program, Michigan State University, East Lansing, Michigan, United States of America; Gettysburg College, United States of America

## Abstract

The vertical zonation patterns of intertidal organisms have been topics of interest to marine ecologists for many years, with interspecific food competition being implicated as a contributing factor to intertidal community organization. In this study, we used behavioral bioassays to examine the potential roles that interspecific aggression and food competition have on the structuring of intertidal hermit crab assemblages. We studied two ecologically similar, sympatric hermit crab species, *Clibanarius digueti* [1] and *Paguristes perrieri* [2], which occupy adjacent zones within the intertidal region of the Gulf of California. During the search phase of foraging, *C. digueti* showed higher frequencies of aggressive behaviors than *P. perrieri*. In competition assays, *C. digueti* gained increased access to food resources compared to *P. perrieri*. The results suggest that food competition may play an important role in structuring intertidal hermit crab assemblages, and that the zonation patterns of Gulf of California hermit crab species may be the result of geographical displacement by the dominant food competitor (*C. digueti*).

## Introduction

Hermit crabs are characteristic organisms found in nearly all marine habitats across the globe [3], and thus their biology has been widely studied. In particular, hermit crabs have commonly been used as subjects in studies addressing resource use and competition. Intertidal hermit crab assemblages offer a unique study system to test hypotheses on the ecological effects of interspecific competition because (1) multiple ecologically similar species often live in close sympatry [3], and (2) sympatric species often show stereotypical patterns of vertical (depth) zonation [4] with species segregating into distinct bands within the intertidal zone [5]. The causes of these zonation patterns in hermit crabs are largely unknown, but like other intertidal organisms [6], [7] are believed to be the result of a complex interplay among abiotic (e.g., desiccation risk, aerial exposure) and biotic (e.g., competition) pressures [8].

Because of the intimate relationship that hermit crabs share with the empty gastropod shells they inhabit [3], the vast majority of research on hermit crabs to date has focused on how they use, and compete for, shell resources by means of aggressive interactions [3], [8]–[13]. As a result, shell selection and shell competition have historically been used by ecologists to explain patterns of hermit crab abundances and distributions within the intertidal zone [3], [8]. However, relatively little is known about how hermit crabs compete for other important ecological resources, and how the outcomes of these competitions influence the distributions and abundances of competing species. In particular, the potential importance of interspecific food competition on the structure of hermit crab assemblages has been largely overlooked aside from a few pioneering studies [14]–[16].

Hermit crabs are generalist detritivores [3], [17], but many species show a strong affinity for consuming carrion (dead or decaying animal tissue), likely because of carrion’s high nutritional value [18] compared to other available food items (e.g., algae). Ecological studies have confirmed that carrion is a particularly scarce resource in the intertidal zone [19], and one that is believed to limit the sizes of scavenger populations [16], [20]. Hermit crabs face intense competition for carrion resources because carrion is (1) distributed irregularly in space and time [19] and (2) often rapidly removed from a system because of being relied upon by numerous competing species [16], [19]–[21]. Indeed, the scarcity of intertidal carrion resources has been shown to limit the population sizes of intertidal scavengers [20]. Thus, it is likely that differences in the competitive abilities for food between sympatric species have important implications for the structure of hermit crab assemblages, including species distributions [5], [22]–[24] and abundances within the intertidal zone [16]. Ecological research has shown that dominant competitors will stake out the most profitable foraging grounds and geographically displace inferior competitors [25]. Thus, the zonation patterns observed among sympatric hermit crab species may be the result of competitive displacement by the dominant food competitor from the most profitable foraging grounds.

Hermit crabs rely on aggressive interactions to settle resource disputes. Differences in aggressive behaviors between species can result in unequal access to resources between competitors, with the outcomes usually favoring the more aggressive species [21], [26]. Thus, it can be predicted that species differing in competitive abilities will also differ in aggressiveness. In this study, we used behavioral bioassays to test the hypotheses that two intertidal hermit crab species occupying adjacent zones within the intertidal region differ in their (1) rates of aggression (both inter- and intraspecific) during the search phase of foraging (i.e., when stimulated to forage but no food is present) and (2) competitive abilities for food. To test these hypotheses, we studied two ecologically similar, sympatric hermit crab species, *Clibanarius digueti* and *Paguristes perrieri*, from the Gulf of California. *C. digueti* is the most abundant hermit crab species in the Gulf of California [27] and generally occupies areas higher in the intertidal zone than *P. perrieri* [4], [28], although distributional overlap between the species is commonly observed [27] (personal observation). Previous studies have suggested that abiotic factors (i.e., desiccation risk) are not strong determinants of the distributions of some intertidal hermit crab species [29], including *C. digueti* and *P. perrieri* [28]. Thus, it is reasonable to suggest that the species may segregate their distributions based on the outcomes of interspecific resource competition. The species are of similar body sizes [28], and recent field observations (Tran, unpublished data) have shown that they overlap in their carrion preferences.

## Materials and Methods

### Ethics Statement

This study used only invertebrate animals, which are not regulated by the Institutional Animal Care and Use Committee at Michigan State University.

### Animals, Housing and Maintenance

Wild-caught *Clibanarius digueti* and *Paguristes perrieri* from the Gulf of California were obtained from a commercial distributor (A & M Aquatics, Lansing, MI) and acclimated to laboratory conditions for a minimum of two weeks prior to use in experiments. During acclimation, animals were held communally in mixed-species groups in 10-gallon glass aquaria containing aerated artificial saltwater (ASW; Instant Ocean) and kept under a 12 h light: 12 h dark cycle. The two species were housed together during acclimation to simulate natural sympatry. This ASW, and all ASW mentioned in this report, was maintained at 24–28°C, pH 8.2–8.4, and specific gravity of 1.022–1.025. Animals were fed 2–3 times per week with a krill meal-based pellet food (NewLife Spectrum). This feeding amount has been used in previous studies [30] and is believed to keep the animals in a healthy physiological state while minimizing the buildup of nitrogenous waste products emanating from uneaten food.

Because previous studies suggest sexual differences in competitive abilities occur in other *Clibanarius* species [31], only male animals were used for aggression and competition experiments. Animals were sexed by visually examining the gonopores. Because of their dark body coloration, *C. digueti* could only be sexed after evacuation from its shell. The shells of *C. digueti* were removed by gently flaming the tip of the shell, causing the animal to evacuate without harm and preserving the integrity of the shell for re-entry. The animals appeared behaviorally normal following this manipulation, and readily re-entered their shells. If any injury or behavioral abnormalities were noted, the animal was not used. *P. perrieri*’*s* light body coloration allowed them to be sexed without removal of the shell by holding the animal inverted under water until the animal emerged from its shell and exposed the gonopores.

Following gender determination, animals were housed in small groups in plastic containers (20×13×14 cm or 26×16×17 cm) containing ASW and a gravel substrate for a minimum of 4 days to allow sufficient time to recover from handling stress. Because we allowed a minimum of 4 days to recover from handling stress, it is highly unlikely that the different methods of sex determination used between species influenced the animals’ behaviors in our experiments. This, however, was not empirically tested. The size of the containers used to house experimental animals had no effect on either species for any of the measured behaviors (Mann-Whitney U-tests, *P*>0.05).

**Figure 1:**
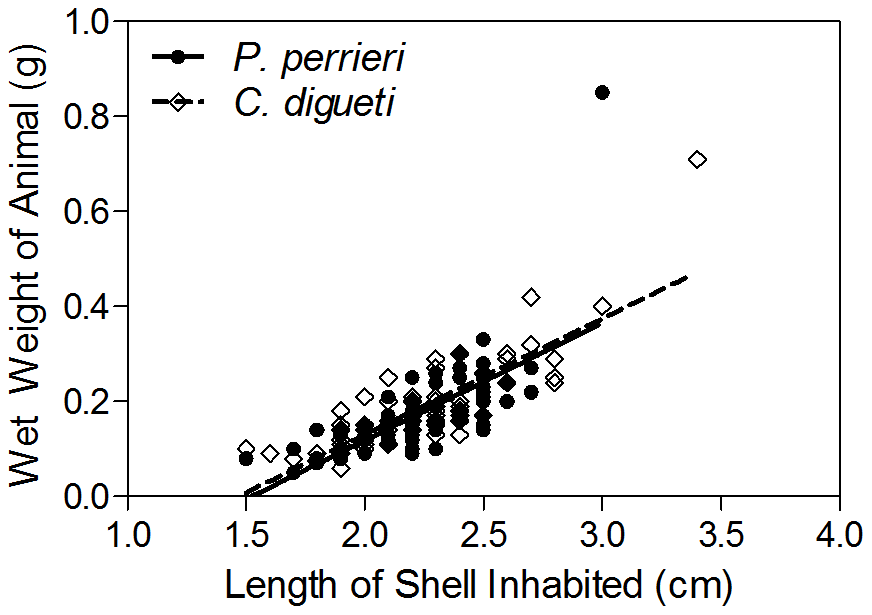
Relationship between wet weight (g) and shell length inhabited (cm). Data were drawn from lab populations of *C. digueti* (*N* = 75) and *P. perrieri* (*N* = 79) following acclimation in housing tanks. Wet weights were measured from animals after removal of their shells. Lines represent best fit lines from linear regression analyses. *C. digueti* best fit line: *y* = 0.2442*x−*0.3584. *P. perrieri* best fit line: *y* = 0.2472*x−*0.3764.

To test aggression and competitive abilities, we sized-matched pairs of animals. These pairs of animals were always drawn from separate plastic housing containers in order to alleviate the effects of any pre-existing dominance hierarchies that may have been formed among tankmates [32]. For all experiments assessing interspecific differences, we housed animals in containers with conspecifics only, and for experiments assessing intraspecific differences, we housed animals in mixed-species containers. On the day the experiments were conducted, experimental pairs of animals were formed by selecting one animal from separate containers. All pairs of animals were size-matched within 3 mm shell length. Size-matching controlled for the effect of body size on aggression and competitive abilities. Shell length was used as a proxy for body size because (1) the species show similar relationships between wet body weight (g) and length (cm) of shell inhabited (Figure 1), (2) during acclimation in housing tanks, animals were provided a plethora of empty shells of different shapes and sizes so they could choose optimally-fitting shells [12], and (3) using animals of equal shell size helped to ensure that aggressive interactions observed during the trials were the result of food competition, and not the result of motivation to switch shells, which could confound the results of these experiments. This method was effective since test crabs rarely showed shell investigation behavior, and only one case of shell switching was documented during the trials. Both species routinely occupied *Cerithium stercusmuscarum* shells. We did not assay for differences in behavior based on shell species occupied.

For experiments assessing feeding times in the absence of a competitor, we used animals of both sexes (11 males: 4 non-gravid females of each species) that had previously been used in other behavioral experiments and were randomly drawn from mixed-species population tanks. We did this because we had a limited availability of *P. perrieri* males and had no *a priori* reason to believe that normal feeding behaviors (1) would be affected by the animals’ previous use in other experiments, or (2) differed between sexes when no competitors were present.

### Testing Apparatus

The testing apparatus consisted of a 250 mL glass Erlenmeyer flask (8 cm bottom diameter) containing 250 mL of ASW and clean, white gravel substrate. This apparatus was large enough that the test animals could remain physically separated and were not forced to interact.

### Food Odor and Food Pellet Preparation

The food odor and food pellets used in this study were made from the animals’ laboratory pellet food because (1) we did not have access to natural carrion sources from the field, (2) the animals were familiar with the food and its odor from exposure during acclimation to the lab, and (3) previous experiments have shown that the odor extracted from the food evokes consistently strong foraging behaviors in both test species [30]. A liquid food extract (FE) was prepared by macerating 2 g of the animals’ normal pellet food diet (NewLife Spectrum) in 200 mL of ASW for 5 minutes. The liquid supernatant was frozen (−4°C) in ∼5 mL aliquots in glass vials and thawed at room temperature immediately prior to use. Food pellets used in “Interspecific Competition in the Presence of Food” experiments were made by macerating 4 g of pellet food (NewLife Spectrum) in 10 mL distilled water until blended. The contents were poured into a 5 cm diameter plastic petri dish and air dried at room temperature (∼22°C) until solid. The solid food pellet was broken into ∼0.5 cm^3^ pieces for use in the experiments. This pellet size was sufficient to allow both competitors to feed simultaneously, but small enough so that the animals would be forced to interact physically if feeding simultaneously.

### Interspecific Aggression in the Presence of Food Odor Only

Animals were given no food for 2 days prior to use to ensure motivation to forage during trials. On the day of the experiment a size-matched pair of heterospecific animals was formed, placed into the testing apparatus, and given a minimum of 15 minutes to acclimate. Following acclimation, 2 mL FE were pipetted into the top of the apparatus using a glass pipette. The animals were allowed 30 seconds to initiate foraging behaviors, after which the numbers of aggressive and submissive behaviors (Table 1) exhibited by each animal were counted for 10 minutes. Only trials in which both animals showed obvious foraging behaviors (e.g., increased locomotion, substrate probing, feeding movements) [33] after the FE was introduced were included in analyses. This ensured that the animals were motivated to forage and in a healthy physiological state during the trials. Twenty trials of this experiment met these criteria and were used for analyses. We compared the number of (1) aggressive behaviors, and (2) submissive behaviors observed during the trials between the species using a two-sided Wilcoxon Sign-Ranks test for matched pairs. Paired analyses were required because the behaviors of one species could directly influence the behaviors of the other species during the trials. These, and all other statistical tests mentioned in this report, employed a significance cutoff of *P = *0.05.

**Table 1:** Aggressive and Submissive Behaviors Observed During Foraging.

Behavior	Description
*Aggressive*	
Approach	Animal moves towards other animal
Display	Animal shows chelipeds and/or legs in threatening move
Attack	Animal strikes other animal with chelipeds/legs
Grasp	Animal grasps onto other animal’s shell
*Submissive*	
Retraction	Animal pulls its body into shell
Retreat	Animal moves away from other animal

### Intraspecific Aggression in the Presence of Food Odor Only

Size-matched pairs of conspecific animals were tested using the same procedure listed in the previous section. One *C. digueti* trial was excluded from analyses because the animals switched shells during acclimation, and we could not be sure that subsequent aggressive behaviors were not shell-related. Seventeen trials for each species were used for analyses (34 trials total). We compared the number of (1) aggressive, and (2) submissive behaviors shown between species during intraspecific trials using a two-sided Mann-Whitney U-test. Paired tests were not required for these statistical analyses because the species were tested independently of each other, and thus the behaviors of one species could not directly affect the behaviors of the other.

### Interspecific Competition in the Presence of Food

Prior to use in these experiments, animals were withheld from food for 1 day prior to testing to ensure motivation to forage (preliminary observations showed 1 day of food deprivation was sufficient for this purpose). Size-matched pairs of heterospecific animals were placed into the testing apparatus and given a minimum of 15 minutes to acclimate. Following acclimation, a single food pellet (∼0.5 cm^3^) was placed an equal distance from both animals and 2 mL FE were immediately pipetted into the testing apparatus to initiate foraging behaviors. FE was used to initiate foraging because (1) previous observations showed that FE exposure caused rapid foraging responses and thus decreased the total time needed to run each trial, and (2) without added water movement the odor emanating from the food would not disperse rapidly through the apparatus. The animals were allowed 2 minutes to locate the food item. Observation time was started either when an animal contacted the food item or 2 minutes elapsed after food placement. The first animal to contact the food item and the total time spent feeding by each animal were recorded for a period of 10 minutes. Feeding was characterized by the animal controlling (grasping) the food item with its chelipeds or legs and picking off small pieces with the chelipeds. Only trials in which (1) both animals showed foraging behaviors after food placement and (2) at least one animal fed were used for analyses. Twenty six trials of this experiment met these criteria and were used in the analyses. During some trials, the pellet was broken after manipulation by the animals, allowing both animals to feed simultaneously on different pieces of the food. Because this could not be controlled, the trials were continued as normal and both animals were considered to be feeding. Qualitative observations showed that *P. perrieri* fed more frequently on broken pieces of food than *C. digueti.* The proportion of trials in which each species was the first to contact the food item was compared using a chi-square goodness-of-fit test. Time spent feeding by each species was compared using a two-sided Wilcoxon Sign-Ranks test for matched pairs.

### Feeding Times without Competition

This experiment was done to determine if feeding times for both species were similar in the absence of a competitor. The same experimental procedure was used as explained in the previous section, except that only a single animal was placed in the apparatus during each trial. Feeding times were compared between species using a two-sided Mann-Whitney U-test.

## Results

### Interspecific Aggression in the Presence of Food Odor Only

During interspecific foraging bouts in the presence of food odor only, *C. digueti* showed significantly more aggressive behaviors than *P. perrieri* (*W = *171.0, *P = *0.0015; Table 2), while *P. perrieri* showed significantly more submissive behaviors than *C. digueti* (*W = *−92.0, *P = *0.0172; Table 2). *C. digueti* frequently initiated the interactions by approaching *P. perrieri*, and *C. digueti* escalated the interactions by frequently attacking and grasping *P. perrieri* (Table 2). *P. perrieri* routinely responded to *C. digueti*’s aggressive behaviors by retracting into their shells (Table 2).

**Table 2:** Behaviors observed during interspecific aggression trials.

Behavior	*Clibanarius digueti*	*Paguristes perrieri*
Approach	53	10
Display	0	2
Attack	21	5
Grasp	46	15
***Total Aggressive***	**120**	**32**
***Mean ± SEM Aggressive***	**6.00±0.73**	**1.60±0.50**
Retreat	4	2
Retract	4	33
***Total Submissive***	**8**	**35**
***Mean ± SEM Submissive***	**0.40±0.17**	**1.75±0.45**

Total counts of behaviors shown. Counts were summed among test animals of the same species. *N* = 20 trials.

### Intraspecific Aggression in the Presence of Food Odor Only

The number of aggressive (*U = *54.0, *P = *0.0019) and submissive (*U = *85.0, *P = *0.0295) behaviors were significantly higher for *C. digueti* than *P. perrieri* during intraspecific foraging bouts (Table 3). The frequencies of all measured behaviors were higher for *C. digueti* than *P. perrieri* (Table 3).

**Table 3:** Behaviors observed during intraspecific aggression trials.

Behavior	*Clibanarius digueti*	*Paguristes perrieri*
Approach	76	30
Display	12	2
Attack	43	23
Grasp	52	20
***Total Aggressive***	**183**	**75**
***Mean ± SEM Aggressive***	**10.76±1.90**	**4.41±1.34**
Retreat	31	9
Retract	19	12
***Total Submissive***	**50**	**21**
***Mean ± SEM Submissive***	**2.94±0.79**	**1.24±0.64**

Total counts of behaviors shown. Counts were summed among test animals of the same species. *N* = 17 trials for each species.

### Interspecific Competition in the Presence of Food

During interspecific food competition, *C. digueti* fed for significantly more time than *P. perrieri* (*W* = 179.0, *P* = 0.0238; Table 4). Differences in feeding times between the species can partly be explained by the fact that *C. digueti* was the first animal to contact and feed on the food item in 20 of the 26 trials analyzed (*χ* ^2^ = 7.54, df = 1, *P* = 0.006).

**Table 4:** Feeding times when species fed together (with competition) and independently (without competition).

Experiment	Measurement	*C. digueti*	*P. perrieri*
With Competition (N = 26)	Mean (SEM)	360.60 (42.04)	234.11 (33.76)
	Median	433.50	170.50
Without Competition (N = 15/species)	Mean (SEM)	268.90 (69.07)	352.50 (71.23)
	Median	135.00	530.00

Mean (SEM) and median feeding times are in seconds.

### Feeding Times without Competition

In the absence of a competitor, the feeding times of *C. digueti* and *P. perrieri* were not significantly different (*U* = 102.0, *P* = 0.6738; Table 4).

## Discussion

The results of this study support the hypotheses that intertidal hermit crab species occupying adjacent zones in the intertidal zone differ in their rates of aggression and competitive abilities for food resources. *C. digueti* was shown to exhibit higher rates of aggression and lower rates of submissive behaviors than *P. perrieri* during interspecific trials. Higher rates of both aggressive and submissive behaviors exhibited by *C. digueti* compared to *P. perrieri* during intraspecific trials suggest that *C. digueti* is generally more interactive than *P. perrieri*. The lack of significant difference in time spent feeding in the absence of a competitor suggests that (1) there was no difference between species in their preferences for the food item used in this study, and (2) the species have similar feeding rates when their feeding is not interfered with by a competitor. Significantly higher feeding times by *C. digueti* compared to *P. perrieri* when feeding together indicate that *C. digueti* is the dominant food competitor and uses aggression to dissuade *P. perrieri* from foraging. The results also suggest that *C. digueti* may gain competitive advantages over *P. perrieri* by locating food resources faster, despite similarities in the species’ chemosensitivities to olfactory foraging cues [30].

The results of this study highlight the need for further research on the feeding ecology of hermit crabs. Although most hermit crabs exhibit generalist diets [3] that can be incredibly diverse in the absence of sympatric competitors [17], intraspecific [14] and interspecific [16], [21] competition have been shown to limit food access to certain individuals. Indeed, recent field analyses of the diets of *C. digueti* and *P. perrieri* suggest that the species diets are differentiated despite overlapping preferences for food items (Tran, unpublished data). These findings, in combination with the results reported in this study and what is known about the generality of hermit crab diets [3], [17], suggest that (1) interspecific competition makes food a potentially limiting resource, and (2) the outcomes of food competition between sympatric hermit crab species may play an important role in structuring hermit crab assemblages.

Because so little is known about food competition in hermit crabs, it is difficult to compare its importance to that of shell competition in influencing species abundances and distributions. It is important to note that food competition is not necessarily independent of shell competition. Because hermit crabs also use aggression to contest for shells [3], [12], [34], it is likely that highly aggressive species would be better at competing for both food and shells than less aggressive species. Thus, it seems unlikely that there would be an ecological trade-off between a species’ ability to compete for food and shells. Additionally, recent research suggests that partially predated or damaged gastropods may serve as both food and shell resources for hermit crabs [35]. Thus, aggression in hermit crabs may facilitate both shell and food resource competition. In nature, both *C. digueti* and *P. perrieri* commonly occupy *Cerithium stercusmuscarum* shells, but are also found inhabiting the shells of other gastropod species (Tran, personal observation). To our knowledge, no data exists on the shell preferences of these species (e.g., dimensions, weight), and thus we cannot comment on whether differences in preferences exist between species or whether these preferences influence species distributions [8].

The results of this study highlight the need for detailed field experiments measuring the importance of food competition in structuring hermit crab assemblages. While we can infer based on our laboratory results that the differences in competitive abilities between *C. digueti* and *P. perrieri* may have important ecological implications, detailed field experiments are needed to determine if the trends observed in the lab hold true in natural settings. It is our hope that the results presented in this manuscript will help raise awareness of the need for field experimentation.

The differences in competitive abilities for food between *C. digueti* and *P. perrieri* may have a number of ecological implications, such as influencing species abundances. *C. digueti* was shown to be the dominant food competitor in this study, and is also the most numerically dominant species in the Gulf of California [27]. McNatty et al. [16] showed that hermit crab (*Coenobita spp.*) population sizes are reduced in areas of overlap with a sympatric food competitor. Thus, it is plausible that the population sizes of *P. perrieri* are limited through competitive inferiority for food. *P. perrieri* may persist in areas of sympatry by outcompeting *C. digueti* for other important resources (e.g., shells) [11], utilizing shells of different architectures than are preferred by *C. digueti* [29], occupying microhabitats that *C. digueti* prefers to avoid [24], [36], or by utilizing less preferred food items.

Differences in competitive abilities for food may also influence the distribution of *C. digueti* and *P. perrieri* within the intertidal zone. In nature, *C. digueti* is found from the subtidal to the high-intertidal (highest abundance in the high-intertidal), while *P. perrieri* is found only in a narrow band in the mid-intertidal (Tran, personal observation). We hypothesize that the zonation patterns of *C. digueti* and *P. perrieri* may be strongly influenced by the outcomes of food competition between the species. Previous studies across habitats and taxonomic groups have shown that dominant competitors often stake out the best habitats for their ecological needs and competitively displace inferior competitors from those habitats [5], [24], [37]–[39]. In the context of our study, this would mean that *C. digueti* stakes out areas higher in the intertidal zone and competitively displaces *P. perrieri.* This competitive displacement explanation is, of course, dependent on food abundance being highest in the upper intertidal zone. While this has not been tested empirically within the natural ranges of these species, it is plausible given that carrion (dead or decaying animal tissue) is often stranded at the land-water interface (i.e., the strand line). As the result of wave action, carrion may be lifted higher in the intertidal zone (closer to the shoreline) where it interacts with the land and becomes stranded [40]. Thus, proximity to the strand line would afford animals more frequent opportunities to encounter carrion resources. Indeed, studies conducted in the Gulf of California suggest that ocean-derived carrion reaches land and is an important aspect of the diet of land animals in the area [40]. Thus, it appears that proximity to the strand line would offer foragers increased access to important food resources. However, detailed field analyses of the carrion distribution in the natural habitats of *C. digueti* and *P. perrieri* are needed to validate this assumption.

We do not intend to suggest that food competition is the only factor influencing the zonation patterns of *C. digueti* and *P. perrieri*. Indeed, the zonation pattern of organisms are often dictated by a complex interplay among biotic and abiotic environmental conditions [5]–[8], [24], [31], [41], and thus can be heavily influenced by local environmental conditions [41]. Generally, it has been shown that the lower limit of a species’ distribution is determined by biotic factors, such as predation pressure and competition for limiting resources [8], [41]. In contrast, the upper limit of a species’ distribution is believed to be determined by abiotic factors, such as temperature, salinity, or desiccation tolerance [6], [7]. Areas higher in the intertidal zone generally expose animals to harsher abiotic conditions than lower intertidal areas due to emersion during periods of low tide [6]. The true extent to which these abiotic factors influence the distributions of intertidal hermit crabs remains unknown. However, three studies lend support to the belief that abiotic factors (i.e., desiccation tolerance) are not strong determinants of the zonation patterns of intertidal hermit crab species [28], [29], [36]. While the *Clibanarius* genus of hermit crabs has been shown to have higher desiccation tolerance than the *Calcinus* genus [36], Gherardi and Nardone [29] found that *Calcinus laevimanus* has a higher intertidal distribution than *Clibanarius humilis.* Similarly, Harvey [28] concluded that desiccation tolerance was not a strong determinant of the positioning of *C. digueti* and *P. perrieri* in the intertidal zone of the Gulf of California. These findings lend support to our hypothesis that the zonation patterns of *C. digueti* and *P. perrieri* may be shaped by the outcomes of interspecific food competition.
